# Quality of life and emotional distress in advanced prostate cancer survivors undergoing chemotherapy

**DOI:** 10.1186/1477-7525-2-37

**Published:** 2004-07-23

**Authors:** Peter C Trask

**Affiliations:** 1 Centers for Behavioral and Preventive Medicine. The Miriam Hospital and Brown Medical School, Coro Building, Suite 500, One Hoppin St. Providence, RI 02903, USA

## Abstract

Prostate cancer continues to occur in over 230,000 men each year. Although the majority of these will be diagnosed in the early stages, there remains a proportion who will either be diagnosed in late stage disease or develop progressive disease. In patients with advanced disease, research has recently focused on using chemotherapy for symptom management and palliation. Given that the focus is not on cure, the effect of chemotherapy on quality of life is of utmost importance. The present article will 1) summarize the current chemotherapeutic studies that have included a quality of life component, with a particular focus on pain and fatigue, 2) discuss the issue of distress in advanced prostate cancer patients treated with chemotherapy, and 3) suggest future research directions.

From the studies that have investigated quality of life, it appears that several chemotherapeutic agents reduce pain and fatigue, although the development of fatigue is often the dose-limiting factor of some agents. The assessment of overall quality of life has occurred in several studies, however, an examination into the impact of chemotherapy on functional status and interpersonal relationships has not been studied. Finally, in contrast to the numerous studies in early stage prostate cancer patients, the presence and effect of distress in chemotherapy-treated prostate patients has not been examined. As such, increased attention is needed to quality of life during phase I-III chemotherapy trials.

## Background and significance

Prostate cancer will be diagnosed in an estimated 230,110 men during 2004 [1]. There will also be approximately 29,900 men who will die from prostate cancer this year, making it the second leading cause of cancer death among men. While early detection and improved treatments have resulted in improved 5-year survival rates for individuals with early stage prostate cancer (recent data have put the 5-year survival rates at 100% for men diagnosed with local and regional prostate cancer), there remains a proportion of men (roughly 14%) who will be diagnosed with advanced prostate cancer. For these individuals, the 5-year survival rate is much lower. Indeed only 34% of men diagnosed with distant disease will survive for 5 years [1].

For men with late stage disease, chemotherapy is increasingly being examined as a treatment option, although the goal is usually palliative in nature and may not extend length of survival [2,3]. Chemotherapy is also being explored as adjuvant therapy in men with early stage disease where length of survival may be lengthened by its administration. In both cases, but particularly among men receiving chemotherapy as treatment for advanced cancer, the effect that chemotherapy may have on quality of life (QOL) is extremely important. This QOL includes not only the individual's physical well-being, but their mental well-being, role functioning and levels of emotional distress as well. At present, few studies have examined the impact of chemotherapy on the physical and emotional QOL of prostate cancer patients. The goals of the current article are to: 1) summarize the current chemotherapeutic studies that have included a quality of life component, with a particular focus on pain and fatigue, 2) discuss the issue of distress in advanced prostate cancer patients treated with chemotherapy, and 3) suggest future research directions.

### Impact of chemotherapy on quality of life

The inclusion of QOL endpoints in chemotherapy trials with cancer patients started in earnest during the last decade, with the majority of studies assessing the impact of chemotherapeutic agents on symptoms such as pain and fatigue. Since then, the inclusion of QOL endpoints has become a more common outcome in chemotherapy studies, although many continue to neglect the psychological component, focusing instead on the occurrence of symptoms and their impact on physical QOL.

## Methods

A comprehensive search of English-language articles in PubMed was conducted to identify studies that had assessed quality of life and/or emotional distress as part of chemotherapeutic trials in advanced cancer patients (Table 1). The keywords "advanced cancer", "chemotherapy", and "quality of life" were included in each search. In subsequent searches "distress", "anxiety", and "depression" were added.

**Table 1:** Summary of reviewed articles

**Authors**	**Chemotherapeutic agent**	**QOL measure**	**Direction of effect**
Tannock et al. (1996)	Mitoxantrone	EORTC QLQ-30; Pain rating	Improved pain, mood & physical activity
Beer et al., (2001)	Docetaxel	Present Pain Intensity scale	Improved pain
Sinibaldi et al. (1999)	Docetaxel	Brief Pain Inventory	Improved pain
Copur et al. (2001)	Docetaxel	Numeric rating scale	Improved pain
Gravis et al. (2003)	Docetaxel	EORTC QLQ-30; Pain VAS	Improved role, social functioning, overall QOL, pain, fatigue, constipation
Small et al. (2000)	Suramin	Brief Pain Inventory	Improved pain
Fuse et al. (1996)	Cisplatin/carboplatin	Rating scale	Improved pain
Kreis et al. (1999).	Docetaxel	Physician-graded events	Presence of fatigue dose-limiting factor
Hamilton et al. (2003)	Vinblastine	Physician-graded events	Presence of fatigue dose-limiting factor
van Andel et al. (2003)	Epirubicin	QLQ-30	Reduced fatigue, transient improvement in emotional, social and cognitive functioning
Kornblith et al. (2001)	Docetaxel	FACT-P, Mental Health Inventory-17	Transient improvements in emotional well-being, improved overall QOL

### Pain

In one of the first studies to examine the impact of chemotherapy on pain, Tannock, Osoba, Stockler et al. [4] randomized 161 hormone-resistant prostate cancer patients either to prednisolone or prednisolone plus mitoxantrone. The goal was to determine the impact on pain reduction as a palliative endpoint. Also included was the overall assessment of QOL using the EORTC QLQ-30 and a specific prostate cancer QOL measure composed of nine analog scales. The results demonstrated that the addition of mitoxantrone to prednisolone reduced pain in 29% of patients compared to 10% for those who only received prednisolone. Improvements in pain, mood, and physical activity were also observed on the QOL measures for the individuals who received mitoxantrone. Additional analyses from this study revealed that after six weeks of treatment, pain and physical functioning remained improved in the mitoxantrone plus prednisone group. Moreover, after 12 weeks of treatment, overall quality of life in this group was improved, as was quality of life in four functional domains and nine specific symptoms [5]. Additional studies with mitoxantrone have demonstrated that up to 40% of patients will report reductions in pain and improvements in QOL [6]. As a result, in hormone-resistant prostate cancer patients, mitoxantrone is believed to be of some benefit [4].

Pain was also assessed in several studies utilizing docetaxel either alone or in combination with estramustine in androgen-independent prostate cancer [7-9]. In one study, the administration of docetaxel as a single agent resulted in pain relief, defined as two-point reduction in the Present Pain Intensity scale on two consecutive evaluations, in 30% of patients [10]. Moreover, an additional 18% reduced their consumption of analgesics by at least 50%. Mean pain scores on quality of life scales were also reduced over the course of treatment [7]. In two studies using the combination of docetaxel and estramustine, pain reductions were observed in 70% and 82% of patients [8,9]. Unfortunately, the methods used to assess pain are not thoroughly reported in either study.

In a recent study, docetaxel was administered intravenously on a daily basis for 6 consecutive weeks followed by a 2-week break between each of up to 4 cycles [11]. The authors assessed QOL prior to the start of chemotherapy, the first day of each treatment cycle, and then 15 and 30 days after the last treatment. At baseline, metastatic, hormone-refractory prostate cancer patients reported decreased QOL in role, social functioning, and overall QOL, as well as pain, fatigue, and constipation. Of note, following the first cycle, all patients reported improved QOL and pain relative to baseline levels. At the end of treatment, pain levels had increased somewhat, but were still lower than baseline levels. Combined, these studies suggest that docetaxel is effective in reducing pain in androgen-independent patients.

Reductions in pain have also been observed in studies using suramin and epirubicin. In a study by Small et al. [12], comparing suramin plus hydrocortisone to a placebo plus hydrocortisone, reduction in pain for longer durations were observed in the suramin treated group. Interestingly, however, more global measures of QOL were not impacted by that treatment. Despite the reduction in pain, because overall QOL and survival rates are similar to hydrocortisone treated patients, whether suramin should be regularly used in the treatment of hormone-refractory prostate cancer patients is unclear. This is also true for suramin with anthracycline regimens, where the combination, because it does not offer significant improvements in disease management, is not recommended to hormone-resistant prostate cancer patients [13-18]. Pain reduction has also been observed in prostate cancer patients treated with epirubicin [19]. Interestingly, the reductions in pain did not correspond to patient-reported improvements in QOL. As a result, like suramin, the authors concluded that epirubicin should not be used as a monotherapy.

The use of the more commonly known platinum compounds (e.g., cisplatin, carboplatin) have also demonstrated beneficial efforts on pain reduction. In advanced prostate cancer patients treated with a carboplatin/epirubicin/etoposide regimen, pain relief was observed in 44% of the patients [20].

The overall point of these studies is that for prostate cancer patients with advanced disease, there are a variety of chemotherapeutic agents that have beneficial palliative effects, specifically pain reduction. Despite this, there is no definitive palliative pain regimen in this population and no defined algorithm for pain management using chemotherapy.

### Fatigue

Several studies have investigated the relationship between chemotherapeutic agents and fatigue in prostate cancer patients. Among the agents studied are the taxanes, specifically docetaxel and paclitaxel. In phase I studies of docetaxel and estramustine, the presence of fatigue has been used to identify the dose-limiting factor [21,22]. Currently, a phase III trial is underway to determine the impact on QOL of docetaxel/estramustine compared with mitoxantrone/prednisolone [23]. In a similar phase I/II study examining the effect of vinblastine for androgen-independent prostate cancer, fatigue was also reported as the dose-limiting factor. More importantly, however, the agent was found to be inactive resulting in the conclusion that vinblastine is not an appropriate treatment [24].

Fatigue was also an endpoint in a well-designed study that assessed the effect of 25 mg/m2 epirubicin administered intravenously on a weekly basis compared to 100 mg/m2 administered every 4-weeks [19]. After four weeks of treatment, individuals who were in the Epi100 group reported less fatigue. They also reported better emotional, social, and cognitive functioning as measured by QLQ-30, although these differences disappeared at repeated assessments. The effect of subsequent administration of either dosage of epirubicin on fatigue was not reported, although the authors note that there were no differences at subsequent assessments between groups. As such, it is unknown whether epirubicin decreases or increases fatigue in prostate cancer patients over time.

### Quality of Life (QOL)

In contrast to the numerous QOL studies of early-stage prostate cancer patients, beyond pain and fatigue, the assessment of QOL in advanced stage prostate cancer patients is relatively lacking. By and large there are no studies assessing the common areas of physical well-being (e.g., urinary and sexual functioning) or psychological well-being (e.g., distress related to treatment or sequelae), and social or family well-being in chemotherapy treated patients. The notable exception is a study of the QOL of 44 hormone refractory prostate cancer patients and their partners by Kornblith et al. [25]. In a phase II study of docetaxel, estramustine, and low dose hydrocortisone, prostate cancer patients were assessed with the Functional Assessment of Cancer Therapy-Prostate, as well as the Mental Health Inventory-17. Over a period of 6-months, men reported significant improvements on the Emotional Well-being subscale. Further examination, however, revealed that when compared to the baseline assessment, emotional well-being was only better at the two and four month assessments. Total FACT-P scores improved over the course of treatment as well, as did prostate cancer-specific concerns, although the former was not significant [25]. The authors concluded that the benefits of the chemotherapeutic regimen were limited to the first four-months of treatment.

### Impact of chemotherapy on emotional distress

In contrast to the various studies that have investigated the presence of emotional distress, depression, and anxiety in early stage prostate cancer patients, there is remarkably little concerning these issues in those with advanced cancer. Indeed, a Pubmed search using the terms "distress AND advanced prostate cancer" resulted in only 12 studies. Of these, only 3 studies specifically focused on advanced prostate cancer [25-27], with 1 of these focusing on physical symptom distress [26] and another focused on a group of asymptomatic men with nonmetastatic prostate cancer receiving androgen deprivation therapy [27]. Only one of the studies, the previously cited Kornblith et al. [25] study assessed distress in chemotherapy treated men. The result, therefore, is an overwhelming lack of information concerning the emotional functioning of chemotherapy-treated prostate cancer patients. It is hard to believe that these individuals who are frequently androgen-deprived, have a short life expectancy, and are being treated with chemotherapy for palliative purposes are not experiencing some emotional distress. Even as part of phase II studies, where the sample sizes are small, emotional distress should be examined if for no other reason than to provide initial information as to what changes may be seen in a larger sample, and what issues need to be assessed.

## Conclusions and implications

Chemotherapy is being examined with greater frequency in the treatment of advanced prostate cancer. Since the goal of treatment for these individuals is more likely to be palliation than cure, understanding the impact on QOL is that much more important. For example, understanding the impact on QOL can assist clinicians and patients in decision-making by providing data on whether symptom benefit obtained outweighs the toxicities from treatment.

Thus far, several studies have identified improvements in pain and fatigue as a result of chemotherapeutic protocols, although in some studies, the presence of fatigue is a dose-limiting factor. Disappointing is the lack of focus on the more global aspects of quality of life, the impact on physical, psychological, social, and family well-being. How the treatment of the individual affects their relationships with others (e.g., marital relationship, sexual functioning) has yet to be studied in this population. Also yet to be studied is the impact on emotional well-being. The presence of distress in cancer patients is well-documented (e.g., [28]), and it is known that early-stage prostate cancer patients exhibit distress (bother) over physical side effects (i.e., urinary and sexual functioning). In addition, distress related to PSA tests has been documented. What then is the emotional well-being of advanced prostate cancers like? Does the administration of chemotherapy play a role in either the development of distress or its reduction? As the continued development and testing of chemotherapeutic medications for advanced prostate cancer moves from phase I through phase II and III trials, it is of ever-increasing importance to assess the impact on QOL and distress. Only through its inclusion will a clearer picture of the efficacy of chemotherapy be observed. Future research can do this by developing protocols that include HRQOL parameters as a standard part of the design process. Both the industry that develops and tests the compound and the academics who deliver it to the patient should be involved in identifying which aspects of QOL are essential to target.
